# Prevalence of chronic kidney disease-associated pruritus, and association with sleep quality among hemodialysis patients in Pakistan

**DOI:** 10.1371/journal.pone.0207758

**Published:** 2018-11-29

**Authors:** Inayat Ur Rehman, Syed Munib, Amutha Ramadas, Tahir Mehmood Khan

**Affiliations:** 1 School of Pharmacy, Monash University, Jalan Lagoon Selatan, Selangor Darul Ehsan, Malaysia; 2 Department of Pharmacy, Abdul Wali Khan University, Mardan, Pakistan; 3 Department of Nephrology, Institute of Kidney diseases, Peshawar, Pakistan; 4 Jeffrey Cheah School of Medicine & Health Sciences, Monash University Malaysia, Jalan Lagoon Selatan, Selangor Darul Ehsan, Malaysia; 5 Institute of Pharmaceutical Sciences (IPS), University of Veterinary & Animal Sciences (UVAS), Lahore, Pakistan; Heart of England NHS Foundation Trust, UNITED KINGDOM

## Abstract

**Background:**

The prevalence of chronic kidney disease-associated pruritus (CKD-aP) varies from 22% to 84% among patients receiving hemodialysis. It occurs more frequently at night, and often affects patient’s sleep quality. CKD-aP is often unreported by patients, and many do not receive effective treatment. There is, however, a paucity of available data on the prevalence and impact of CKD-aP on patients receiving hemodialysis in Pakistan.

**Methods:**

A multicenter cross-sectional study was undertaken from July 2016 to April 2017 at a tertiary care hospitals in Pakistan.

**Results:**

354 patients undergoing hemodialysis were studied. 35.6% had CKD for 1–2 years, and 42.4% were receiving hemodialysis for 1–2 years. The prevalence of pruritus was 74%. The median [interquartile range] score for pruritus was 10.0 (out of possible 25) [8.0–12.0]; while the median [interquartile range] Pittsburgh Sleep Quality Index (PSQI) score was 8.0 (out of possible 21) [7.0–10.0]'. Pruritus was significantly correlated with the sleep score (r = 0.423, p<0.001). The results of the multivariate linear regression revealed a positive association between pruritus and age of patients (β = 0.031; 95% CI = 0.002–0.061; p = 0.038) and duration of CKD (β = -0.013; 95% CI = -0.023 –-0.003; p = 0.014). Similarly there was a positive association between sleep score and duration of CKD (β = 0.010; 95% CI = 0.002–0.019; p = 0.012) and pruritus (β = 0.143; 95% CI = 0.056–0.230; p = 0.001).

**Conclusions:**

Chronic kidney disease-associated pruritus is very common in patients receiving hemodialysis in Pakistan. Pruritus is significantly associated with poor sleep quality.

## Background

Pruritus is a troublesome complication which affects the health-related quality of life (HRQOL) of patients with end-stage renal disease (ESRD) [1]. Chronic kidney disease-associated pruritus (CKD-aP) is an undesirable condition that triggers itching, and negatively affects sleep quality [2]. It has been reported that the prevalence of pruritus ranges from 40–70% [3–5], while sleep disorders due to CKD-aP ranges from 28.8–90% [6–10]. CKD-aP has been found to result in nocturnal awakenings and difficulty in falling asleep [4, 11]. Indeed patients on hemodialysis suffering from moderate to extreme pruritus were found to be three times more likely to have poor quality of sleep [12] In a study done by Yamada et al., pruritus was reported in 66.9% of hemodialysis patients, and of these, 41.2% had pruritus-related sleep disorders [13]. It has also been observed that patients with poor sleep quality caused by moderate to extreme pruritus, have a higher risk of mortality [14]. In Stage 5 CKD patients, 61% experienced difficulty falling asleep because of pruritus, while 44% experienced sleep disruptions due to itchy sensations [4]. In Pakistan, the prevalence of CKD-aP ranges from 64.64–77.7% [15, 16]. Pisoni et al found that the prevalence of moderate to severe pruritus due to CKD was 45% in the Dialysis Outcomes and Practice Patterns Study (DOPPS) I (1996–2001), while in the DOPPS II (2002–2004) it was 42%, however, 45% of patients experienced poor quality of sleep [12]. Poor quality of sleep in turn has been linked to an increased risk of hypertension [17], CKD-aP [18, 19], impaired glucose tolerance [20], diabetes mellitus [21], depression [22], impaired HRQOL, increased mortality rate [23], and increased healthcare utilization [24].

Ample research has been undertaken to study sleep quality among CKD patients undergoing hemodialysis [7, 12, 25], however, there is very limited research so far which has sought to study the impact of CKD-aP on the sleep quality of patients undergoing hemodialysis in Pakistan. As such, the aim of this research was to determine the prevalence of CKD-associated pruritus, and the association of CKD-aP with the sleep quality of patients having CKD-aP on hemodialysis in Pakistan.

## Methodology

A multicenter cross-sectional study was undertaken to estimate the burden and impact of pruritus on the sleep quality of CKD-aP among patients undergoing hemodialysis from July 2016 to April 2017 at tertiary-care hospitals, namely the Institute of kidney diseases, Peshawar and the Pakistan Kidney Patients Association, Rawalpindi Pakistan.

### Participants

#### Inclusion criteria

Patients aged 18 years and above, of both genders, undergoing haemodialysis, who understand the Urdu language, and were willing to participate in the research project were included.

#### Exclusion criteria

Patients not fulfilling the above criteria were excluded from the study.

### Sample size

The sample size was calculated using a confidence interval of 95%, and 5% precision. By using a prevalence rate of pruritus in Pakistan of 70%, which was obtained from our pilot study [26], and assuming the following: p = 0.7, Z = 1.96 (for 95% level of confidence), and d = 0.05; a sample size of 322 was calculated. The following formula for sample size[27] was used, which is well established in literature.

### Procedure

Patients were approached while they were undergoing haemodialysis. The objectives of the study were explained, and signed informed-consent forms were obtained from those who were willing to participate. Data was collected using validated versions of the questionnaires in the Urdu language; i.e. the Urdu 5D-itch scale and the Urdu version of the Pittsburgh sleep quality index (PSQI).

Baseline data collected included demographic data, socioeconomic status, duration of CKD, duration of hemodialysis, comorbid conditions, medicine used, and laboratory data. The 5D-itch scale developed by Elman et al [28] measures duration, degree, direction, disability, and distribution. The duration, degree, and direction domains contain one item each, while the disability domain have four items to assess the effect of itching on daily activities, and its score was calculated by selecting the highest score. In the distribution domain, the body parts affected by pruritus can be selected, and as such participants could select as many parts as they wished. A scoring bin was used for the body parts affected by pruritus, where 0–2 body parts were scored as 1; 3–5 body parts were scored as 2, 6–10 body parts were scored as 3, 11–13 body parts were scored as 4, and 14–16 body parts were scored as 5. The overall score of the 5D-itch scale was calculated by summing all the five domains, where a score of 5 indicates no pruritus, and the score of 25 indicates severe pruritus[26]. The PSQI developed by Buysse et al. [29] measures self-rated sleep quality over the past one month (30 days), and consists of 19 items combined for 7 component scores, including subjective sleep quality, sleep latency, sleep duration, habitual sleep efficiency, sleep disturbances, use of sleep medication, and daytime dysfunction. The overall score was combined by summing up the scores of these 7 components. The PSQI score ranges from 0 to 21. The participants were then classified into two groups according to their PSQI score: poor-sleep group (PSQI > 5) and good-sleep group (PSQI ≤ 5). Higher scores are indicative of poor and impaired sleep quality.

### Statistical analysis

Data were analysed using the Statistical Package for Social Sciences version 20.0 (SPSS Inc., Chicago, IL). Normality was assessed using the Kolmogorov–Smirnov test. In order to represent continuous variables, median and interquartile range [IQR] “25th" and “75th" percentile were used, whereas, for representing categorical variables, numbers and frequencies were used. Associations between categorical variables were analysed using Pearson chi-square tests, whereas associations between continuous variables were assessed using Spearman’s rho. Associations between categorical and continuous variables were assessed using the Mann–Whitney U-test. These potential predictor variables were chosen on the basis of statistical significance from univariate analysis. The variables with a univariate p-value <0.20 were subjected to multivariate analysis [30]. The use of a univariate p value<0.20 has the advantage of tending to include more variables in a multivariate analysis, while traditional levels of p-value such as 0.05 can fail in identifying variables known to be important. Non-parametric tests like the Kruskal-Wallis test, and Mann-Whitney tests, were used and bivariate analysis was done to estimate the association between pruritus with sleep quality. A p-value of less than 0.05 indicated statistical significance.

## Results

**Fig 1:**
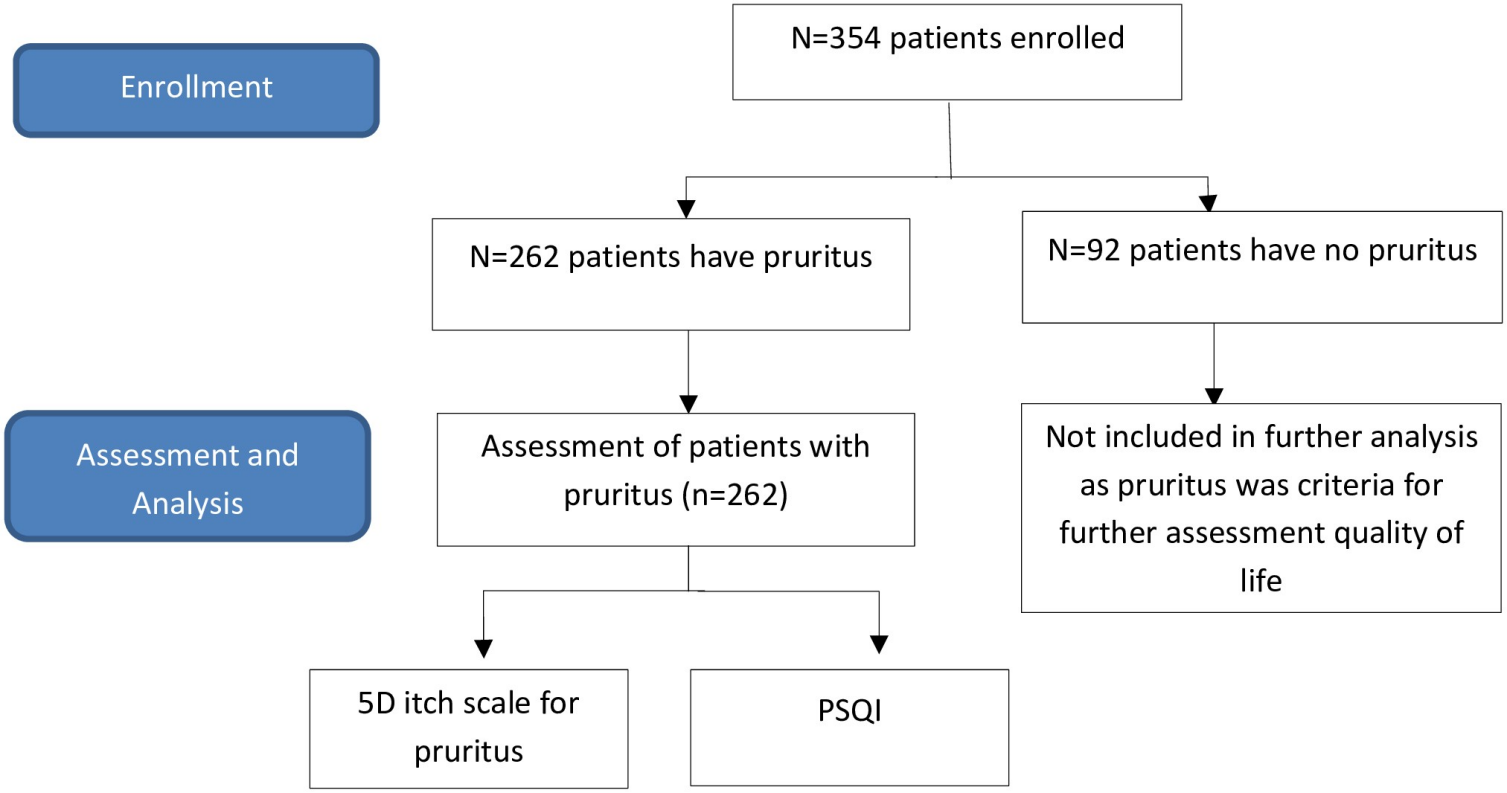
Study flow diagram.

A total of 354 patients were recruited (response rate = 100%). The flow on how patients were recruited is shown in (Fig 1). The majority were male (66.1%) and the median [IQR] age of patients was 42.0 [34.0–50.0]. The age of patients, duration of having CKD and being on dialysis was significantly higher in patients with pruritus. Hypertension (n = 215), diabetes mellitus (n = 47), hyperlipidemia (n = 25) and cardiovascular diseases (n = 7) were the most common co-morbidities observed in CKD patients with pruritus (Table 1). Laboratory parameters are shown in (S1 Appendix).

**Table 1:** Demographics of participants (n = 354).

	Total no. of patient (n = 354) N (%)	Without pruritus n = 92 N (%)	With pruritus n = 262 N (%)	p-value
**Gender**				
*Male*	234 (66.1)	58 (24.8)	176 (75.2)	0.471 a
*Female*	120 (33.9)	34 (28.3)	86 (71.7)	
**Median age [IQR] in years**	42.0 [34.0–50.0]	40.0 [30.0–48.0]	42.0 [35.0–51.0]	0.013* b
*18–30 years*	64 (18.1)	27 (42.2)	37 (57.8)	0.012* a
*31–40 years*	108 (30.5)	24 (22.2)	84 (77.8)	
*41–50 years*	99 (28)	26 (26.3)	73 (73.7)	
*51–60 years*	57 (16.1)	9 (15.8)	48 (84.2)	
*61–70 years*	19 (5.4)	3 (15.7)	16 (84.2)	
*70 years and above*	7 (2)	3 (42.9)	4 (57.1)	
**Median duration of CKD [IQR] in months**	36.0 [12.0–48.0]	24.0[12.0–36.0]	36.0 [24.0–48.0]	<0.001*^b^
*< 1 year*	40 (11.3)	22 (55.0)	18 (45)	<0.001* a
*1–2 years*	126 (35.6)	40 (31.7)	86 (68.3)	
*3–4 years*	114 (32.2)	15 (13.2)	99 (86.8)	
*5–6 years*	37 (10.5)	10 (27.0)	27 (73.0)	
*7–8 years*	20 (5.6)	2 (10.0)	18 (90)	
*9–10 years*	9 (2.5)	1 (11.1)	8 (88.9)	
*11 years and more*	8 (2.3)	2 (25.0)	6 (75.0)	
**Median duration on hemodialysis [IQR] in months**	24.0 [12.0–36.0]	7.0 [2.25–12.0]	24.0 [12.00–36.0]	<0.001*^b^
*< 1 year*	86 (24.3)	51 (59.3)	35 (40.7)	<0.001* a
*1–2 years*	150 (42.4)	31 (20.7)	119 (79.3)	
*3–4 years*	97 (27.4)	10 (10.3)	87 (89.7)	
*5–6 years*	13 (3.7)	0	13 (100)	
*7–8 years*	8 (2.3)	0	8 (100)	
**frequency of hemodialysis**				
*Twice a week*	354 (100)	92 (26)	262 (74)	-
**Presence of co-morbidities** **				
Diabetes mellitus	47	11 (23.4)	36 (76.6)	
Hypertension	215	38 (17.7)	177 (82.3)	
Hyperlipidemia	25	7 (28)	18 (72)	
Cardiovascular diseases	7	2 (28.6)	5 (71.4)	
Gout	-	-	-	
Goiter	-	-	-	

^**a**:^ Chi-square test;

^**b**:^Mann-Whitney U test;

*p<0.05.

** (Diabetes mellitus, hypertension, hyperlipidemia) were the most common comorbidities observed in current cohort of patients.

Figures were >100% as patients may be suffering from more than one chronic condition.

Among the 262 patients with pruritus, responses on the 5D-itch scale revealed that 64.5% experienced pruritus for a duration of less than 6 hours a day, 53.4% experienced mild pruritus, while 28.6% respondents claimed that their pruritus is much better but still present. In the disability domain for sleep disturbance, 30.9% indicated that pruritus occasionally delayed them being able to fall asleep, 53.8% claim that pruritus never affected their leisure/social activities, and 56.1% noted that pruritus never affected their housework/errands. Among the sixteen body parts, the back, palm, head/scalp, abdomen, and thighs, were the most affected among respondents. The scoring bin designed for the distribution domain showed that the majority of respondents (49.6%) fall in Score bin 1 as shown in (Table 2). In order to determine the score of pruritus, domains of the scale were totaled. Most of patients (52.0%) had mild pruritus. The overall pruritus score with median [IQR] was 10.0 [8.0–12.0] as shown in (Table 2).

**Table 2:** Responses by respondents on Urdu 5D itch scale (n = 262).

Statement	N (%)
**Duration**	
*Less than 6hours/day*	169 (64.5)
*6–12 hours/day*	58 (22.1)
*12–18 hours/day*	27 (10.3)
*18–23 hours/day*	3 (1.1)
*All day*	5 (1.9)
**Degree**	
*Not present*	31 (11.8)
*Mild*	140 (53.4)
*Moderate*	70 (26.7)
*Severe*	15 (5.7)
*Unbearable*	6 (2.3)
**Direction**	
*Completely resolved*	106 (40.5)
*Much better but still present*	75 (28.6)
*Little bit better but still present*	70 (26.7)
*Unchanged*	9 (3.4)
*Getting worse*	2 (0.8)
**Disability: Sleep**	
*Never affects sleep*	49 (18.7)
*Occasionally delays falling asleep*	81 (30.9)
*Frequently delays falling asleep*	76 (29)
*Delays falling asleep and occasionally wakes me up at night*	33 (12.6)
*Delays falling asleep and frequently wakes me up at night*	23 (8.8)
**Disability: Leisure/Social**	
*Never affect activity*	141 (53.8)
*Rarely affects activity*	46 (17.6)
*Occasionally affects activity*	49 (18.7)
*Frequently affects activity*	24 (9.2)
*Always affects activity*	2 (0.8)
**Disability: Housework/Errands**	
*Never affect activity*	147 (56.1)
*Rarely affects activity*	52 (19.8)
*Occasionally affects activity*	45 (17.2)
*Frequently affects activity*	12 (4.6)
*Always affects activity*	6 (2.3)
**Disability: Work/School**	
*Never affect activity*	175 (66.8)
*Rarely affects activity*	38 (14.5)
*Occasionally affects activity*	35 (13.4)
*Frequently affects activity*	12(4.6)
*Always affects activity*	2 (0.8)
**Distribution**	
*Head/Scalp*	67 (25.8)
*Face*	44 (17)
*Chest*	44 ((17)
*Abdomen*	62 (23.8)
*Back*	118 (45.6)
*Buttocks*	46 (17.6)
*Thighs*	50 (19.3)
*Lower legs*	41 (15.8)
*Sole*	30 (11.6)
*Palm*	81 (31)
*Tops of Hands/Fingers*	46 (17.6)
*Forearm*	30 (11.5)
*Upper arm*	39 (15)
*Points of Contact w/ Clothing (e*.*g*. *waistband/undergarments)*	51 (19.6)
*Groin*	40 (15.4)
*Tops of Feet/Toe*	48 (18.5)
**Distribution bin score**	
*Score bin 1*	130 (49.6)
*Score bin 2*	104 (39.7)
*Score bin 3*	21 (8)
*Score bin 4*	2 (0.8)
*Score bin 5*	5 (1.9)
**Total score of 5D itch scale (ranged from 5–25)**	
Median [IQR]	10.0[8.0–12.0]
5–10 (Mild pruritus)	136 (52.0)
11–19 (Moderate pruritus)	125 (47.7)
20–25 (severe pruritus)	1 (0.3)

With regard to severity of pruritus, the majority (56.5%) of respondents were having moderate pruritus, followed by 34.4% with mild pruritus, and 9.1% with severe pruritus.

To quantify the severity of sleep disturbances among CKD patients undergoing hemodialysis, the Pittsburgh sleep quality index (PSQI) was used. With regard to the time that respondents went to bed in the past one month, 63% reported 9 pm—11 pm, while as it pertained to the time taken by respondents to fall asleep each night during the past month, 52.7% reported 15–30 min. As for the actual hours of sleep each night during the past month, 51.5% had an actual sleep time of 5–6 hours. The factors cited as responsible for troubled sleep during the past month as reported by respondents are shown in (Table 3). A lower score indicates healthier sleep quality, while a score of 5 or greater is indicative of “poor sleep quality”.

**Table 3:** Responses on PSQI by respondents on Urdu PSQI questionnaire (n = 262).

Statement	N (%)
During the past month, what time have you usually gone to bed at night	
*7pm–9 pm*	52 (19.8)
*9 pm–11 pm*	165 (63)
*11 pm–1 am*	42 (16)
*1 am–4*.*30 am*	3 (1.1)
During the past month, how long (in minutes) has it usually takes you to fall asleep each night	
*< 15 min*	39 (14.9)
*15–30 min*	138 (52.7)
*30–60 min*	75 (28.6)
*> 60 min*	10 (3.8)
During the past month, what time have you usually gotten up in the morning	
*12am–2 am*	12 (4.6)
*2 am–4 am*	28 (10.7)
*4 am–6am*	155 (69.2)
*6am–8am*	51 (19.5)
*8am–10am*	16 (6.1)
During the past month, how many hours of actual sleep did you get at night	
*> 7 hours*	41 (15.6)
*6–7 hours*	54 (20.6)
*5–6 hours*	135 (51.5)
*<5 hours*	32 (12.2)
During the past month, how often have you had trouble sleeping because	
a. Cannot get to sleep within 30 minutes	
*Not during the past month*	69 (26.3)
*Less than once a week*	101 (38.5)
*Once or twice a week*	36 (13.7)
*Three or more times a week*	56 (21.4)
b. Wake up in the middle of the night or early morning	
*Not during the past month*	91 (34.7)
*Less than once a week*	79 (30.2)
*Once or twice a week*	33 (12.6)
*Three or more times a week*	59 (22.5)
c. Have to get up to use the bathroom	
*Not during the past month*	95 (36.3)
*Less than once a week*	118 (45)
*Once or twice a week*	22 (8.4)
*Three or more times a week*	27 (10.3)
d. Cannot breathe comfortably	
*Not during the past month*	128 (51.1)
*Less than once a week*	65 (24.8)
*Once or twice a week*	16 (6.1)
*Three or more times a week*	47 (17.9)
e. Cough or snore loudly	
*Not during the past month*	134 (51.1)
*Less than once a week*	65 (24.8)
*Once or twice a week*	16 (6.1)
*Three or more times a week*	47 (17.9)
f. Feel too cold	
*Not during the past month*	176 (67.2)
*Less than once a week*	45 (17.2)
*Once or twice a week*	8 (3.1)
*Three or more times a week*	33 (12.6)
g. Feel too hot	
*Not during the past month*	159 (60.7)
*Less than once a week*	53 (20.2)
*Once or twice a week*	10 (3.8)
*Three or more times a week*	40 (15.3)
h. Had bad dreams	
*Not during the past month*	195 (74.4)
*Less than once a week*	40 (15.3)
*Once or twice a week*	9 (3.4)
*Three or more times a week*	18 (6.9)
i. Have pain	
*Not during the past month*	73 (27.9)
*Less than once a week*	104 (39.7)
*Once or twice a week*	31 (11.8)
*Three or more times a week*	54 (20.6)
j. Other reason(s), please describe	
*Not during the past month*	235 (89.7)
*Less than once a week*	24 (9.2)
*Once or twice a week*	2 (0.8)
*Three or more times a week*	1 (0.4)
k. How often during the past month have you had trouble sleeping because of this	
*Not during the past month*	141 (53.8)
*Less than once a week*	54 (20.6)
*Once or twice a week*	20 (7.6)
*Three or more times a week*	47 (17.9)
How would you rate your sleep quality overall	
*very bad*	13 (5)
*Fairly bad*	98 (37.4)
*Fairly good*	131 (50)
*Very good*	20 (7.6)
During the past month, how often have you taken medicine to help you sleep (prescribed or over the counter)	
*Not during the past month*	216 (82.4)
*Less than once a week*	27 (10.3)
*Once or twice a week*	5 (1.9)
*Three or more times a week*	14 (5.3)
During the past month, how often have you had trouble staying awake while driving, eating meals, or engaging in social activity	
*Not during the past month*	203 (77.5)
*Less than once a week*	42 (16)
*Once or twice a week*	4 (1.5)
*Three or more times a week*	13 (5)
During the past month, how much of a problem has it been for you to keep up enough enthusiasm to get things done	
*No problem at all*	32 (12.2)
*Only a very slight problem*	113 (43.1)
*Somewhat of a problem*	93 (35.5)
*A very big problem*	24 (9.2)
Do you have a bed partner or room mate	
*No bed partner or room mate*	64 (24.4)
*Partner/roommate in other room*	43 (16.5)
*Partner in same room*, *but not same bed*	52 (20)
*Partner in same bed*	103 (39.1)
If you have a roommate or bed partner, ask him/her how often in the past month you have had	
a. Loud snoring	
*Not during the past month*	215 (82.1)
*Less than once a week*	36 (13.7)
*Once or twice a week*	3 (1.1)
*Three or more times a week*	8 (3.1)
b. Long pauses between breaths while asleep	
*Not during the past month*	178 (68.2)
*Less than once a week*	52 (19.9)
*Once or twice a week*	3 (1.1)
*Three or more times a week*	28 (10.7)
c. Legs twitching or jerking while you sleep	
*Not during the past month*	178 (68.5)
*Less than once a week*	46 (17.7)
*Once or twice a week*	11 (4.2)
*Three or more times a week*	27 (9.6)
d. Episodes of disorientation or confusion during sleep	
*Not during the past month*	196 (74.8)
*Less than once a week*	38 (14.5)
*Once or twice a week*	6 (2.3)
*Three or more times a week*	22 (8.4)
e. Other restlessness while you sleep	
*Not during the past month*	188 (72.9)
*Less than once a week*	36 (14)
*Once or twice a week*	10 (3.9)
*Three or more times a week*	28 (9.2)

The median and interquartile range [IQR] of the PSQI component scores of sleep duration, sleep disturbance, sleep latency, daytime dysfunction, sleep efficiency, self-account of overall sleep quality, and global scores are shown in (Table 4). Majority of respondents had a higher global PSQI score, with a median and interquartile range [IQR] of 8.00 [7.00–10.00], as shown in (Table 4).

**Table 4:** Distribution of the Pittsburgh sleep quality index (PSQI) scores (n = 262).

Components of PSQI	PSQI sub-component	Frequency	Percentage	Median [IQR]
PSQI component of sleep duration	*> 7 hours*	41	15.6	2.00 [1.00–2.00]
	*6–7 hours*	54	20.6	
	*5–6 hours*	135	51.5	
	*<5 hours*	32	12.2	
PSQI component of sleep disturbances	*0*	2	0.8	1.00 [1.00–2.00]
	*1*	185	70.6	
	*2*	67	25.6	
	*3*	8	3.1	
PSQI component of sleep latency	*0*	19	7.3	1.00 [1.00–2.00]
	*1*	131	50	
	*2*	80	30.5	
	*3*	32	12.2	
PSQI component of daytime dysfunction	*0*	27	10.3	1.00 [1.00–1.00]
	*1*	177	67.6	
	*2*	46	17.6	
	*3*	12	4.6	
PSQI component of sleep efficiency	*>85%*	121	46.2	1.00 [0.00–1.25]
	*75–84%*	76	29	
	*65–74%*	38	14.5	
	*<65%*	27	10.3	
PSQI component of sleep quality	*Very good*	13	5	2.00 [1.00–2.00]
	*Fairly good*	98	37.4	
	*Fairly bad*	131	50	
	*Very bad*	20	7.6	
PSQI component of sleep medication	*Not during the past month*	216	82.4	0.00 [0.00–0.00]
	*Less than once a week*	27	10.3	
	*Once or twice a week*	5	1.9	
	*Three or more times a week*	14	5.3	
**Global PSQI score**	**Median [IQR]**	**8.00 [7.00–10.00]**		

To assess and determine the association between pruritus and sleep score, bivariate analysis with Spearman’s rho was used. Findings revealed a statistically significant weak positive correlation between pruritus with sleep quality scores, where the poor sleep quality increases with an increase in pruritus scores (r = 0.423, p = <0.001). Upon further analysis univariate analysis was performed to identify the potential factors independently associated with pruritus score and the potential factors were chosen on the basis of statistical significance having p-value <0.20 for multivariate analysis. Among eleven factors in the univariate analysis only six had p-value <0.20; (Table 5). The multivariate analysis revealed that there is a positive association between pruritus and age of patients (β = 0.031; 95% CI = 0.002–0.061; p = 0.038) and duration of CKD (β = -0.013; 95% CI = -0.023 –-0.003; p = 0.014).

**Table 5:** Association of chronic kidney disease-associated pruritus with demographics (n = 262).

	Unadjusted	p-value	95% CI	Adjusted	p-value	95% CI
	B			B		
**Gender** ^a^	-0.117	0.780	-0.939; 0.705			
**Age** ^a^	0.030	0.046*	0.000; 0.060	0.031	0.038*	0.002; 0.061
**Duration of CKD** ^b^	-0.014	0.012*	-0.024; -0.003	-0.013	0.014*	-0.023; -0.003
**Duration of dialysis** ^b^	0.017	0.061	-0.001; 0.036	0.016	0.084	-0.002; 0.034
**Hypertension** ^a^	0.660	0.114	-0.160; 1.481	0.628	0.121	-0.168; 1.424
**Diabetes** ^a^	0.663	0.244	-0.455; 1.782			
**Elevated cholesterol** ^a^	-0.042	0.957	-1.568; 1.484			
**WBC** ^a^	0.850	0.218	-0.505; 2.204	0.979	0.149	-0.352; 2.310
**Hb** ^a^	-0.342	0.575	-1.541; 0.857			
**Na** ^a^	0.493	0.198	-0.259; 1.244	0.573	0.126	-0.162; 1.308
**K** ^a^	0.389	0.252	-0.278; 1.056			

Multivariate linear regression was applied. R^2^ = 0.077. Assumptions were fulfilled. Ref:

^**a**:^Gender: Female = 0 and male = 1; hypertension: No = 0, Yes = 1; diabetes: No = 0, Yes = 1; elevated cholesterol: No = 0, Yes = 1; WBC: 0 = < than 5,000/mL, 1 = 5,000–10,000/mL, 3 = > 10,000 /mL; Hb: 0 = < 12 g/dL, 1 = 12–18 g/dL; Na: 0 = < 135 mEq/L, 1 = 135–145 mEq/L, 3 = >145 mEq/L; K: 0 = <3.5 mEq/L,1 = 3.5–5.0 mEq/L, 3 = > 5.0 mEq/L.

^**b**:^ Age (in years) duration of CKD (in months); duration of dialysis (in months) were continuous;

*p<0.05.

**Abbreviations**: WBC: white blood count, Hb: Hemoglobin, Na: Sodium, K: Potassium

Univariate analysis was performed to identify the potential factors independently associated with sleep score and the potential factors were chosen on the basis of statistical significance having p-value <0.20 for multivariate analysis. Among eight factors in the univariate analysis only three had p-value <0.20; (Table 6). The multivariate analysis revealed that there is a positive association between sleep score and duration of CKD (β = 0.010; 95% CI = 0.002–0.019; p = 0.012) and pruritus (β = 0.143; 95% CI = 0.056–0.230; p = 0.001).

**Table 6:** Association between PSQI score with gender, duration of CKD, duration of receiving dialysis, and severity of pruritus (n = 262).

Variables	Unadjusted		p-value	95% CI	Adjusted		p-value	95% CI
	B	Std. Error			B	Std. Error		
**Gender** ^a^	0.174	0.308	0.573	-0.433; 0.781	-	-	-	-
**Age** ^b^	-0.006	0.011	0.596	-0.028; 0.016	-	-	-	-
**Duration of CKD** ^b^	0.012	0.005	0.021*	0.002; 0.021	0.010	0.004	0.012*	0.002; 0.019
**Duration of dialysis** ^b^	-0.003	0.008	0.678	-0.019; 0.012	-	-	-	-
**Hypertension** ^a^	0.407	0.307	0.187	-0.198; 1.012	0.379	0.299	0.206	-0.210; 0.968
**Diabetes** ^a^	0.36	0.425	0.398	-0.478; 1.198	-	-	-	-
**Elevated cholesterol** ^a^	-0.32	0.564	0.571	-1.430; 0.791	-	-	-	-
**Pruritus score** ^b^	0.147	0.045	0.001*	0.057; 0.236	0.143	0.044	0.001*	0.056; 0.230

**Multivariate linear regression was applied**. R^2^ = 0.079. Assumptions were fulfilled.

^a^Gender: Female = 0 and male = 1; hypertension: No = 0, Yes = 1; diabetes: No = 0, Yes = 1; elevated cholesterol: No = 0, Yes = 1

^**b**^Age (in years), duration of CKD (in months), duration of dialysis (in months),

*p<0.05.

The results show there was a statistically significant difference between groups as determined by the Kruskal-Wallis test, where the sleep score median and [IQR] score was higher in respondents suffering from moderate and severe pruritus. Furthermore, post hoc tests to test pairwise comparisons was conducted and results revealed that mild pruritus was statistically different to moderate pruritus (p = 0.001) and severe pruritus (p = 0.001), while moderate pruritus and severe pruritus were not statistically different (p = 0.167). However, the Mann-Whitney test was used to assess the association between sleep scores and gender, and results revealed no statistical difference between both genders (p = 0.385).

## Discussion

CKD-aP is one of the most frequent complications observed in hemodialysis patients. In previous studies, the prevalence rates of CKD-aP among hemodialysis patients differed, ranging from 22% to 84% [31–33]. The prevalence rate of CKD-aP in this study was 74%, however in our pilot study done to validate the 5D-itch scale in Urdu, we found a prevalence rate of 70% in Pakistan [26], which appeared to be almost similar with previous studies done in Pakistan i.e. 64% [34], 64.64% [16], and 77.7% [15]. The severity of CKD-aP, as reported in our findings, shows that 56.5% of respondents were having moderate pruritus, followed by 34.4% with mild pruritus, and 9.2% with severe pruritus; however, our pilot study revealed that 14% had mild pruritus, 50% had moderate pruritus, while 14% had severe pruritus. [26]. Yet another study in Pakistan reported almost similar patterns of pruritus severity with 50%, 28%, and 14% of respondents having mild, moderate, and severe pruritus, respectively [35]. Other cross-sectional studies in Turkey also showed a high prevalence of 85.4% [36], and 53.4% [37], respectively. However, 50.4% of patients experienced moderate pruritus, and pruritus led to sleep disturbances in 33.8% of patients [37]. In our study, a longer duration of CKD and hemodialysis had a very negligible effect on pruritus, which differs from another study in which a duration of hemodialysis of more than 2 years resulted in higher rates of pruritus (75% vs 25%) compared to those doing hemodialysis for less than 2 years [38]. The variation in pruritus prevalence reported in different studies may be due differences in study designs, selection of participants (e.g., study populations, races, and sample sizes), and the definition of CKD-aP [39]. In our study it was also revealed that hemodialysis machines were limited, and pressure from too many patients was a contributing factor to inadequate hemodialysis. Similar findings were reported by Chauhan et al in India, which has a similar socioeconomic status as Pakistan, and which also faces the same problem of increasing patient numbers and insufficient hemodialysis machines [40]. The European guidelines recommend a minimum of three hemodialysis sessions per week totaling at least 12 hour with i.e. Kt/V of at least 1.2 each session (where K is dialyzer clearance of urea, t is dialysis time, and V is volume of distribution of urea) [41]. A higher Kt/V has been associated with reduced pruritus in the majority of observational studies [5, 42, 43].

In our study, pruritus was slightly higher in females compared to males. However other studies found that males were associated with a 1.1 greater adjusted odds of having moderate to severe pruritus [12], and males were associated with a 1.5 greater adjusted odds of having moderate or severe pruritus [7, 44]. Comorbid conditions are also associated with CKD-aP, where in several studies, diabetes mellitus [45] and hypertension [44] were found to be associated with pruritus. Similarly, in our study patients with hypertension and diabetes had higher scores for pruritus.

CKD-aP has been associated with a poor quality of sleep. In our study, 53.4% of patients had moderate sleep disturbance, while 8.4% had severe sleep disturbance. Our findings are aligned with findings from other studies such as DOPPS by Pisoni et al, where more than 45% of patients with moderate to severe pruritus had poor sleep quality [12], while in another study by Narita et al, 70% of patients with severe pruritus, and 34% of patients with mild or moderate pruritus complained of sleep disturbance [7]. Sleep disturbance caused by pruritus was found in 56.5% of patients, with difficulty falling asleep reported by 8.7% patients; while 13.0% were awakened by pruritus, and 34.8% reported symptoms both while falling asleep and on awakening [46]. In another cross-sectional study by Tessari et al, 59.1% of patients experiencing pruritus complained of difficulty in sleeping [47]. Similarly, in a study done by Weiss et al, more than half of hemodialysis patients reported difficulties with falling asleep more than once a week due to itch/pruritus, and of these, 54.5% complained about the quality of sleep, with the findings suggesting that impaired quality of sleep was significantly associated with pruritus, but not with its severity [48]. CKD-aP is associated with poor sleep quality. In our study findings, a statistically significant weak positive correlation was observed between pruritus score with sleep quality score. Mathur et al [5] demonstrated a statistically significant relationship and 20% decrease in uremic pruritus intensity was sufficient to produce a significant improvement in sleep quality[5]. Similarly, Tessari et al in Italy found that pruritus was associated with a statistically significant and dramatic increase in poor sleep [47]. A significant positive correlation between sleep score and pruritus score intensity was also reported by Adejumo et al [25].

### Strength and limitations

The strength of this study is that it is the first such study in Pakistan to assess the association between CKD-aP on the sleep quality of hemodialysis patients. The other strength of the study is that it is a multicenter study involving patients from two centers in different provinces of Pakistan. In our study findings only two confounders showed positive and statistically significant association that’s why the R^2^ value is less and represents only 7% of the population. We could not include the laboratory values for serum calcium, phosphate, magnesium and parathyroid hormone as our study was not funded and these tests are not normally done in the country setting, so we could not find the association.

## Conclusion

Overall, the findings of this study showed that the prevalence of CKD-aP is high among hemodialysis patients. CKD-aP has a negative impact on sleep quality among hemodialysis patients having CKD-aP, and showed positive associations with poor sleep quality.

### Ethical approval and consent to participate

Ethical approval was obtained from the Monash University Human Research and Ethics Committee (MUHREC Approval No: CF16/1766–2016000890). The Institute of Kidney Diseases (IKD Peshawar) Pakistan ethical committee approved this study (Approval No: 844), as well as the ethical committee of the Pakistan Kidney Patient Association (Rawalpindi) Pakistan. Written informed consent was obtained from the participants.

## Supporting information

S1 Appendix Laboratory parameters for respondents (n = 262). (DOCX)
