# Supplementary material for: Prevalence of chronic kidney disease-associated pruritus, and association with sleep quality among hemodialysis patients in Pakistan
Source: PLoS One. 2018 Nov 29;13(11):e0207758. doi: 10.1371/journal.pone.0207758 (PMC6264857; doi:10.1371/journal.pone.0207758)
Supplement: S1 Appendix — (DOCX) [file pone.0207758.s001.docx]

**S1 Appendix:**

**Laboratory parameters for respondents (n=262)**

| **Lab parameters** | **Mean** | **Standard deviation** |
| --- | --- | --- |
| serum creatinine (M =53-105µmol/L) | 802.21 | 358.29 |
| serum creatinine ( F=44-97 µmol/L) | 772.00 | 293.88 |
| Sodium [Na] (135-145) | 136.88 | 11.26 |
| Potassium [K] (3.5-5) | 4.85 | 0.87 |
| Chloride [Cl] | 100.31 | 15.45 |
| Magnesium [Mg] (0.65-1.25mmol/L) | 1.78 | 0.50 |
| White blood cells (5-10) | 7.22 | 1.88 |
| Hemoglobin (12-18) | 10.95 | 9.09 |
| Platelet (150-400) | 253.41 | 64.60 |
| Lymphocytes (25-33%) | 23.49 | 5.57 |
| Monophils (3-7%) | 7.10 | 2.95 |
| Esophils (1-3%) | 2.98 | 1.51 |
